# Safety of robotic hepatic parenchymal transection using scissor hepatectomy and alternative techniques: a cohort study

**DOI:** 10.1007/s00464-025-12382-0

**Published:** 2025-11-14

**Authors:** Elisabeth Miller, Ali Kassem, Nadir Nasir, Erik Rasbach, Moritz Schwab, Jan Heil, Dorothée Sturm, Marko Kornmann, Nuh N. Rahbari, Emrullah Birgin

**Affiliations:** Department of General and Visceral Surgery, Ulm University Hospital, Albert-Einstein-Allee 23, 89081 Ulm, Germany

**Keywords:** Da Vinci®, Liver transection, Minimally invasive liver surgery, Parenchymal dissection, Posthepatectomy complications

## Abstract

**Background:**

Parenchymal transection represents a critical and challenging step in liver surgery. To date, there is no broadly accepted parenchymal transection technique in robotic liver surgery. This study aimed to compare the scissor hepatectomy technique to other parenchymal transection techniques.

**Methods:**

A prospective database comprising 243 consecutive patients who underwent robotic hepatectomies at a single center was reviewed. The cohort was divided based on whether they received robotic parenchymal transection via scissor hepatectomy or alternative transection techniques. Propensity score matching and logistic regression analyses were carried out.

**Results:**

Between 2020 and 2024, a total of 207 patients met the eligibility criteria with a median age of 64 years (55—70). Of these, 117 (57%) patients underwent parenchymal transection by scissor hepatectomy, while alternative transection techniques (including Bipolar, SynchroSeal, VesselSealer, Hydrojet, and CUSA) were utilized in 90 (43%) patients. SH was associated with lower intraoperative blood loss in both unmatched and matched groups (71 patients in each group). Postoperative Grade III or higher morbidity was comparable between groups (13% vs. 14%, *p* < 0.99). No risk factors were identified as being associated with Grade III or higher postoperative morbidity.

**Conclusion:**

Scissor hepatectomy is demonstrated to be a safe pure robotic parenchymal transection technique. Prospective randomized trials are warranted to compare this approach to other parenchymal transection techniques.

**Graphical abstract:**

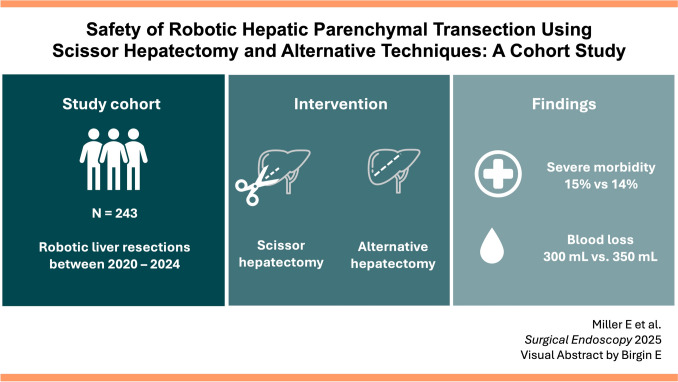

**Supplementary Information:**

The online version contains supplementary material available at 10.1007/s00464-025-12382-0.

Robotic liver surgery has emerged as a transformative advancement of minimally invasive surgery and gained increasing popularity among hepatobiliary surgeons [1–3]. The robotic platform addresses technical limitations of conventional laparoscopy such as restricted degrees of freedom of straight instruments and constrained working angles in liver surgery [4, 5]. However, there is still uncertainty about the optimal technique of robotic parenchymal transection owing to limited robotic instruments for parenchymal transection compared to laparoscopic devices.

The parenchymal transection phase bears a considerable risk of blood loss and bile leakage, which may lead to significant postoperative morbidity [6–8]. Accordingly, various parenchymal transection techniques have been employed in the past two decades in order to minimize postoperative morbidity, including robotic and hybrid laparoscopic devices [9–12]. These techniques involve basic robotic instruments (scissors, bipolar forceps), robotic sealing devices, robotic ultrasonic energy, or hybrid laparoscopic instruments with ultrasound aspiration or hydrodissection technology [12]. The scissor hepatectomy (SH) technique is a pure robotic approach using basic robotic instruments (monopolar scissors and bipolar forceps) enabling an easily reproducible approach for parenchymal transection [13] as previously reported by our group. Initially, we described the SH technique in a clinical series of 39 patients with favorable perioperative outcomes [14]. Nevertheless, a comparative analysis to other available parenchymal transection techniques including larger study cohorts has not been performed so far.

The aim of the present study was to analyze the postoperative outcomes comparing the SH with alternative robotic parenchymal transections (AH) techniques in patients undergoing robotic hepatectomy for benign or malignant liver lesions.

## Methods

### Patients and study design

All patients undergoing robotic hepatectomy for benign or malignant liver lesions from November 2020 through December 2024 at the University Hospital Ulm were identified from a prospectively maintained database. The cohort included consecutive patients (18 years or older) with various minimally invasive parenchymal transection techniques. Cases were excluded if they had a conversion to open surgery (i.e., without any robotic or laparoscopic parenchymal transection), and those involving two-staged hepatectomies, ALPPS (associating liver partition and portal vein ligation for staged hepatectomy) procedure or combined vascular resections with reconstructions (e.g., advanced perihilar cholangiocarcinoma). In addition, patients scheduled for liver biopsies or those who were primarily treated for a liver abscess were also excluded. A written informed consent was obtained before study inclusion. This cohort study was reported in accordance with the STROBE (Strengthening the Reporting of Observational Studies in Epidemiology) statement [15]. The completed STROBE checklist is provided in the supplementary material.

### Surgical technique

All robotic hepatectomies were performed on the daVinci Xi® surgical system (Intuitive Surgical, Sunnyvale, CA, USA) as described previously [16, 17]. Briefly, patients were positioned in a reversed Trendelenburg position, and a pneumoperitoneum between 12 and 18 mmHg was established [18]. Intraoperative ultrasound was utilized for resection guidance. The technique of parenchymal transection was determined intraoperatively at the surgeon’s discretion, utilizing either a robotic or laparoscopic-assisted approach. All procedures were performed by highly experienced minimally invasive liver surgeons. The robotic parenchymal transection involved either one of the following techniques: the scissor hepatectomy technique, employing monopolar scissors and bipolar forceps [13, 14]; the use of a vessel sealing device such as the VesselSealer [19] or the SynchroSeal [20]); or a double bipolar technique [21]. A laparoscopic-assisted parenchymal transection was carried out with a high-pressure water jet (Hydrojet) [22, 23] or ultrasonic parenchymal transection with the CUSA (Cavitron Ultrasonic Surgical Aspirator) device [24]. Intrahepatic vessels and biliary structures were divided using titanium clips (Braun) or Hem-o-lok clips (WECK). Major pedicles and hepatic veins were divided using Endo Gia™ Stapler (Covidien) or the robotic stapling system (Intuitive Surgical). Vascular inflow (Pringle maneuver) was used at the surgeon’s discretion during parenchymal transection. Abdominal drains were not routinely placed.

### Outcomes

Patient demographics included age, sex, body mass index (BMI), the American Society of Anesthesiologists (ASA) score classification, Charlson Comorbidity Index (CCI), cardiovascular comorbidities, diabetes and renal insufficiency, histopathological presence of liver steatosis or cirrhosis, and Child–Pugh score. The extent of hepatectomy was based on the location of the liver lesion and surgeon’s discretion, ranging from partial hepatectomy (non-anatomical resection) to extended right or left hepatectomy, while a parenchyma-sparing approach was primarily intended. The Brisbane nomenclature and the Tokyo 2020 terminology update were applied to classify liver resections [25, 26]. Anatomical liver resections followed Couinaud’s segmentation for complete portal territory removal [26, 27]. Major hepatectomy were those involving resection of more than three anatomical liver segments. The IWATE score was used to assess resection difficulty [28]. Pathological characteristics were extracted from pathology reports, with a negative resection margin defined as malignant tumor cells being more than 1 mm away from the resection margin. Postoperative complications were graded using the Comprehensive Complication Index [29] and Clavien–Dindo classification [30], with severe complications defined as grade IIIa and above. Specific complications after liver resections followed definitions from the International Study Group of Liver Surgery (ISGLS) [31–33].

The primary outcome was the 90-day morbidity rate in the scissor hepatectomy (SH) and alternative hepatectomy (AH) groups.

### Statistics

Descriptive statistics were presented as mean (standard deviation, SD) or median (interquartile range, IQR) for continuous variables with normal or non-normal distributions and frequencies (percentage, %) for categorical variables. Categorical variables were compared using the Chi-Square test or Fisher’s exact test. The Mann–Whitney *U* test was used to analyze continuous variables. A univariate logistic regression analysis was performed to investigate potential risk factors associated with clinically relevant postoperative complications. The risk factors analyzed herein included type of transection technique, type of hepatectomy, extent of parenchyma loss, extrahepatic resections, IWATE score, age, BMI, ASA score, and intraoperative blood loss. Risk factors with a p value < 0.2 were included in a multivariable logistic regression analysis.

Propensity scores for receiving SH were calculated via multivariate logistic regression using pre-treatment variables: age, gender, ASA, BMI, CCI, cirrhosis, diagnosis, major hepatectomy, IWATE score, lesion size, previous liver resection. Variables after parenchymal transection were not included in the model. Patients who underwent SH were matched 1:1 with AH patients using nearest neighbor matching algorithm (caliper width 0.2 SD, without replacement) [17]. The effectiveness of the matching process was evaluated by comparing standardized differences of the covariates across groups, with a standardized difference below 0.2 indicating adequate balance. A subgroup analysis was performed to compare benchmark outcomes in low-risk patients (defined as ASA < 3, absence of major comorbidity, no prior liver resections, and Child A cirrhosis criteria), consistent with a prior benchmark analysis of robotic hepatectomy by extent (major, minor hepatectomy), and difficulty of resection (low, intermediate, expert) [34].

Statistical significance was set at a *p* value of < 0.05. All analyses were performed using R version 4.4.3 (R Foundation for Statistical Computing, Vienna, Austria).

## Results

### Patient characteristics

**Fig. 1:**
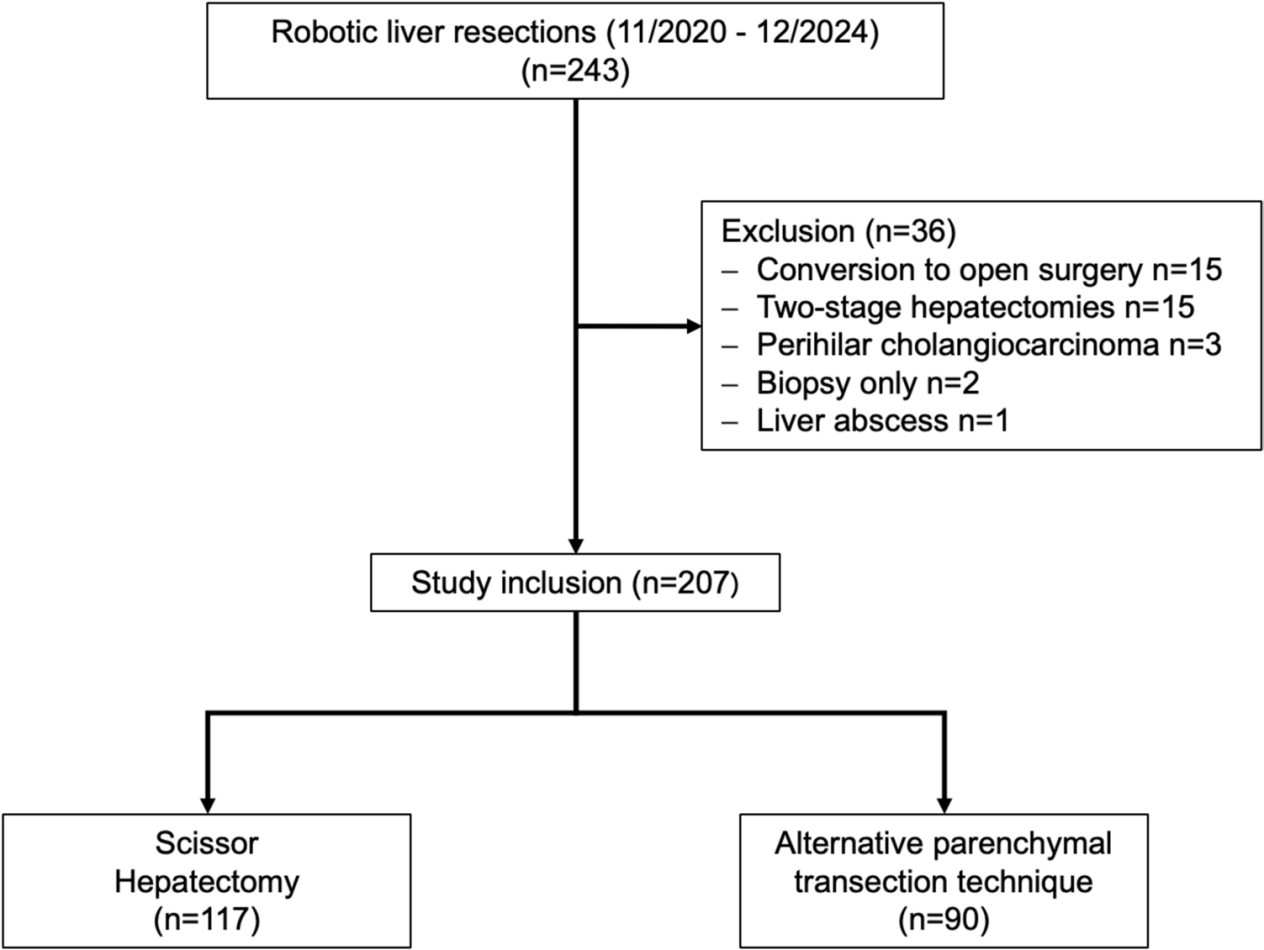
Study flowchart

Of 243 robotic hepatectomies performed during the study period, a total of 207 patients met the inclusion criteria for analysis which includes 117 patients in the SH and 90 patients in the AH group (Fig. 1). A total of 15 cases were excluded owing to intraoperative conversion to open surgery, comprising 1 case in the SH group and 14 cases in the AH group. The indications for conversion were primarily extensive adhesions or oncological considerations. Notably, no conversions were performed because of hemorrhage during parenchymal transection. In the AH group, a pure robotic parenchymal transection was predominantly carried out utilizing the SynchroSeal (*n* = 52, 58%), the VesselSealer (*n* = 17, 19%), and the double bipolar technique (*n* = 15, 17%).

Most patients were male (*n* = 111, 54%) with a median age of 64 years (IQR 55 $-$ 70) and median BMI of 26 kg/m^2^ (IQR 23 $-$ 28) (Table 1). Most liver resections were performed due to malignancies (*n* = 119, 57%), conversely, alveolar echinococcosis was the major indication for benign lesions (*n* = 44, 21%). A high frequency of patients underwent abdominal surgeries prior to hepatectomy (*n* = 115, 56%). Of these, 61 patients (29%) had a history of open abdominal surgery including 17 patients with previous open liver surgery. Liver cirrhosis was present in 41 patients (20%) of the total cohort. The IWATE score was comparable between groups. Baseline characteristics of the SH and AH cohorts were similar, except for a significantly higher Charlson Comorbidity Index (median 6 vs. 4, $p$=0.038) in the SH group. Notably, Child–Pugh class B liver cirrhosis was only present in the SH group (*n* = 10 vs. *n* = 0, *p* = 0.017), and the lesion diameter was significantly smaller in the SH group compared to the AH group (30 mm (IQR 15 $-$ 58 mm) vs. 43 mm (IQR 27 $-$ 64), $p$=0.01).

**Table 1:** Baseline demographics of patients undergoing robotic liver resection using either scissor hepatectomy (SH) or alternative transections techniques (AH)

Characteristics	SH *n* = 117	AH *n* = 90	*p* value
Age, years	64 (56–71)	64 (54–69)	0.414
BMI, kg/m^2^	26 (23–28)	26 (22–29)	0.888
Sex ratio, male:female	66:51	45:45	0.438
ASA			0.268
I	3 (3)	2 (2)	
II	28 (24)	32 (36)	
III	80 (68)	54 (60)	
IV	6 (5)	2 (2)	
Charlson comorbidity index	6 (2–7)	4 (2–6)	**0.038**
Cardiovascular comorbidities	59 (50)	39 (43)	0.383
Renal Insufficiency	10 (9)	3(3)	0.153
Diabetes	28 (24)	12(13)	0.082
Previous abdominal surgery	62 (53)	53 (59)	0.481
Liver steatosis	45 (38)	25 (28)	0.144
Liver cirrhosis	28 (24)	13 (14)	0.128
Child–Pugh class			**0.017**
Child A	18 (15)	13 (14)	
Child B	10 (9)	0	
Diagnosis			0.117
HCC	28 (24)	15 (17)	0.269
CCC	4 (3)	10 (11)	**0.047**
Metastases (CRLM and NCRLM)	34 (29)	28 (31)	0.868
Benign	51 (44)	37(41)	0.829
Maximum lesion size diameter, mm	30 (15–58)	43 (27–64)	**0.01**

Values are presented as median (interquartile range, IQR) or number (percentage), as appropriate. Statistical comparisons were made using the Mann–Whitney *U* test for continuous variables and the Chi-square or Fisher’s exact test for categorical variables. Values in bold, *p* < 0.05

*SH* scissor Hepatectomy, *AH* alternative hepatectomy technique, *ASA* American Society of Anesthesiologists, *BMI* body mass index, *CCC* cholangiocellular carcinoma, *CRLM* colorectal liver metastases, *HCC* hepatocellular carcinoma, *IQR* interquartile range, *NCRLM* non-colorectal liver metastases

### Intraoperative outcomes

The frequencies of patients who underwent partial, non-anatomical hepatectomies were similar in both SH ($n$=55, 47%) and AH ($n$=41, 46%) cohorts (Table 2). Accordingly, minor hepatectomies were the most frequent procedures in both groups. Intraoperative blood loss was significantly lower in the SH group compared to the AH group (300 mL (IQR 100 $-$ 500 mL) vs. 375 mL (IQR 213 $-$ 700 mL), *p* < 0.001). No differences in the rates of extrahepatic resections and operating time were detected.

**Table 2:** Operative details of patients undergoing robotic liver resection using either scissor hepatectomy (SH) or alternative transection techniques (AH)

Characteristics	SH *n* = 117	AH *n* = 90	*p* value
Main hepatectomy procedure			< 0.99
Non-anatomic resection	55 (47)	41 (46)	
Segmentectomy (1 segment)	23 (20)	18 (20)	
Bisegmentectomy (2 segments)	21 (18)	16(18)	
Multiple segments (> 2, e.g., central hepatectomy)	3 (3)	3 (3)	
(Extended) right hepatectomy	10 (8)	8 (9)	
(Extended) left hepatectomy	5 (4)	4(4)	
Extent of liver resection			0.688
Major hepatectomy (> 3 segments)	16 (14)	15 (17)	
Minor hepatectomy (1–3 segments)	101 (86)	75 (83)	
Type of liver resection			0.544
Anatomic	44 (38)	30 (33)	
Non-anatomic	55 (47)	41 (46)	
Both	18 (15)	19 (21)	
Difficulty score (IWATE^a^)	6 (3–10)	5 (4–8)	0.449
Extrahepatic resection	18 (15)	7 (8)	0.147
Blood loss, mL	300 (100–500)	375 (213–700)	** < 0.05**
Operative time, min	199 (136–269)	188 (150–269)	0.813

Values are presented as median (interquartile range, IQR) or number (percentage), as appropriate. Statistical comparisons were performed using the Mann–Whitney *U* test for continuous variables and the Chi-square or Fisher’s exact test for categorical variables. Values in bold, *p* < 0.05

^a^Iwate difficulty scoring system is based on tumor location, tumor size, proximity to major vessels, liver function, and hand-assisted hybrid procedures

*SH* scissor hepatectomy, *AH* alternative hepatectomy technique, *IQR* interquartile range

### Postoperative outcomes

The postoperative outcomes are summarized in Table 3. Overall, a total of 61 patients (29%) experienced postoperative complications, of whom 30 cases (14%) were graded as clinically relevant. A total of 12 patients (6%) had posthepatectomy bile leakage and were treated with postoperative percutaneous interventional drains and endoscopic papillotomy. Three of these 12 patients presented a persisting bile leakage and were revised surgically, specifically, two revisions were performed laparoscopically with draining of the bilioma after anatomical central hepatectomy in one case and non-anatomical resections of the segments IVa/b, V, and VIII in the other case, while the third patient had a defect at the common bile duct which was treated via laparotomy. Three patients had posthepatectomy hemorrhage and were transfused with more than 2 units of packed red blood cells. Among these patients, two underwent surgical revision to drain abdominal hematomas at the resection sites. Posthepatectomy liver failure was diagnosed in three patients. Five patients died within 90 days after surgery during the study period. Two of these deaths were attributed to cardiac heart failure resulting from postoperative decompensation of pre-existing heart failure. Two patients with liver cirrhosis died due to posthepatectomy liver failure following right hepatectomy. Additionally, one patient with cholangiocarcinoma was initiated for palliation treatment due to early progressive disease. No death was related to the type of parenchymal transection technique.

**Table 3:** Postoperative outcomes of patients undergoing robotic liver resection using either Scissor Hepatectomy (SH) or alternative transection techniques (AH)

Characteristics	SH *n* = 117	AH *n* = 90	*p* value
Postoperative complications^b^	33 (28)	28 (31)	0.449
Grade I	8 (7)	11 (12)	
Grade II	8 (7)	4 (4)	
Grade IIIa	10 (9)	6 (7)	
Grade IIIb	5 (4)	2 (2)	
Grade IVa	0	2 (2)	
Grade V	2 (2)	3 (3)	
Clinically relevant complications			< 0.99
< Grade III	16 (14)	15 (17)	
≥ Grade IIIa	17 (15)	13 (14)	
Posthepatectomy bile leakage^c^			
Grade B	6 (5)	3 (3)	< 0.99
Grade C	1 (1)	2 (2)	0.585
Posthepatectomy hemorrhage^c^			0.721
Grade B	0	1(1)	
Grade C	1 (1)	1 (1)	
Posthepatectomy liver failure^c^			< 0.99
Grade B	1 (1)	0	
Grade C	1 (1)	1(1)	
Length of stay, d	5 (4–7)	6 (4–7)	0.526
Readmission within 90 days after surgery	14 (12)	7 (8)	0.449
R Stage			0.538
R0	112 (96)	84 (93)	
R1	5 (4)	6 (7)	

Values are presented as median (interquartile range, IQR) or number (percentage). Statistical comparisons were performed using the Mann–Whitney *U* test for continuous variables and the Chi-square or Fisher’s exact test for categorical variables

^b^Postoperative complications are graded according to the Clavien–Dindo classification [30]. ^c^Posthepatectomy complications as bile leakage, hemorrhage, and liver failure are defined and graded according to the International Study Group of Liver Surgery (ISGLS) [32–34]

*SH* Scissor Hepatectomy, *AH* alternative hepatectomy technique, *IQR* interquartile range, *R stage* resection margin status (R0: no residual tumor, R1: microscopic residual tumor)

There were no significant differences in postoperative outcomes among the study groups regarding morbidity, length of stay, and readmission rate. The mean comprehensive complication index was 8.4 $\pm$ 18 in the SH and 9.6 $\pm$ 21 (*p* = 0.664) in the AH group (Supplementary Fig. 1). The most frequent complication was the presence of an intraabdominal fluid collection (*n* = 15, 13% versus *n* = 10, 11% *p* = 0.837) in both the SH and AH groups. A negative resection margin was achieved in *n* = 196 (95%) patients, while 5 patients with positive resection margins had R1 vascular resections.

### Propensity score matching

After propensity score matching, each study group included 71 patients, with well-balanced covariates between the groups (Supplementary Table 1). There were no differences in the distribution of type of hepatectomy (*p* = 0.794) and technical difficulty as determined by the IWATE score (*p* = 0.900). Blood loss was found to be lower after SH, while the rate of overall morbidity (25% vs. 31%, *p* < 0.698) and grade III or higher (13% vs. 14%, *p* < 0.99) morbidity rates remained comparable between groups. Of note, other postoperative outcomes did not differ between groups.

### Comparison of SH to international benchmarks

A total of 26 patients in the SH group met the criteria of low-risk patients according to international benchmark selection criteria for further subgroup analysis. Low-risk patients were within the cut-off values compared to robotic hepatectomy outcomes in literature except for slightly higher values of intraoperative blood loss in the benchmark subgroups (Supplementary Table 2) [34].

### Analysis of risk factors for postoperative complications

**Fig. 2:**
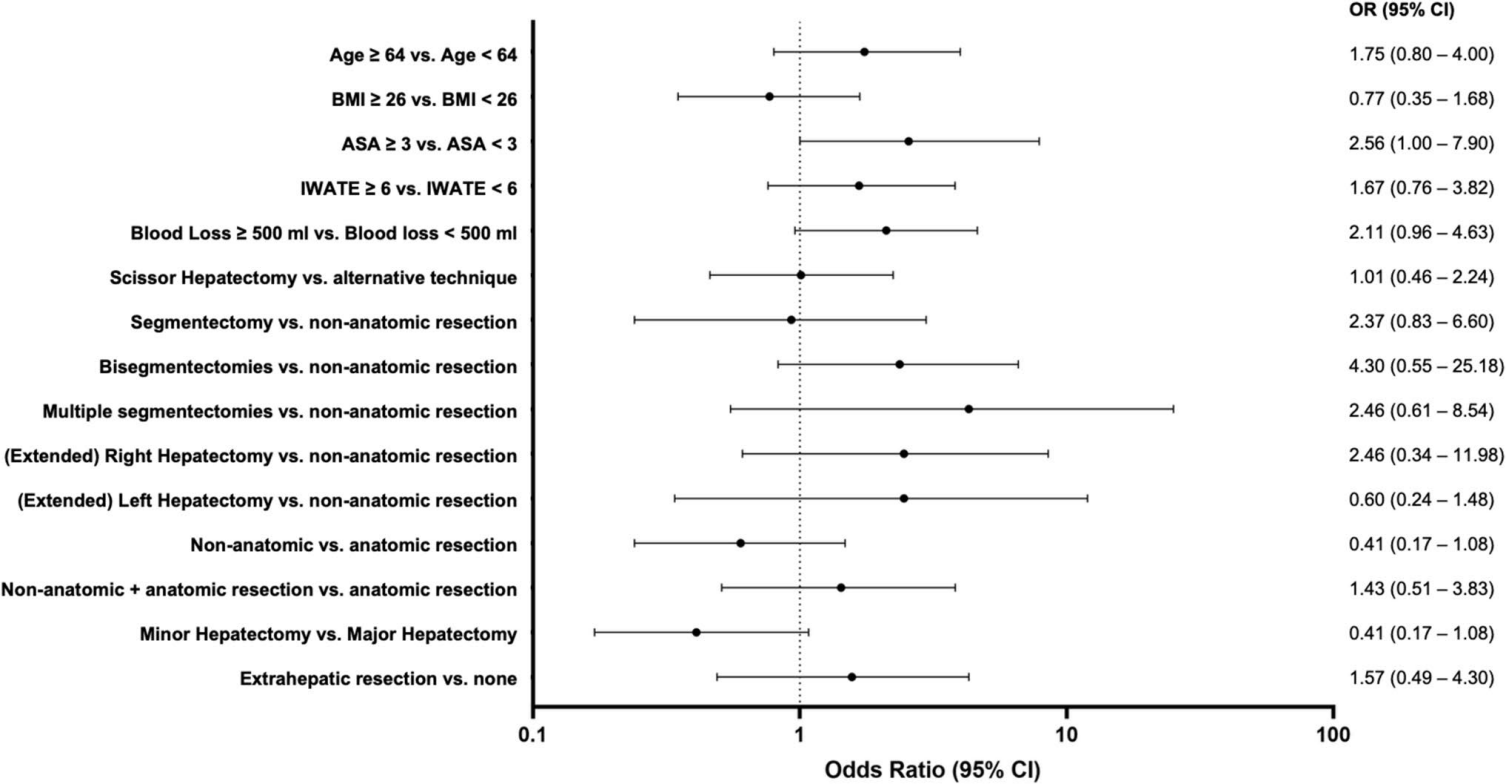
Forest plots were generated to visualize the univariate associations between patient- and procedure-related factors and 90-day postoperative morbidity. Each subplot presents the odds ratio (OR) and 95% confidence interval (CI) for a given variable. *OR* odds ratio, *CI* confidence interval, *ASA* American Society of Anesthesiologists

We analyzed potential risk factors associated with clinically relevant postoperative complications in the entire study cohort using logistic regression analysis. Four preoperative variables (age, BMI, ASA score, difficulty score) and six intraoperative variables (transection technique, type of liver resection, extent of parenchyma loss, anatomical resections, extrahepatic resections, blood loss) were examined using univariate analysis. No specific risk factors including the type of parenchymal transection technique were found to be associated with clinically relevant complications as shown in Fig. 2. In a next step, a multivariable analysis was performed including all risk factors with a *p* value < 0.2 on univariate analysis (Supplementary Table 3). We found no risk factors independently associated with clinically relevant postoperative complications.

## Discussion

This is the first comparative study of the SH and AH techniques for robotic hepatectomies. Our findings revealed no differences in perioperative morbidity and mortality within 90 days after surgery. Most notably, the outcomes for posthepatectomy complications such as bile leakage, hemorrhage, and liver failure were comparable between groups as were the outcomes for operating time and pathological resection margins. Although there was slightly less blood loss in the SH group, the clinical significance of this difference is uncertain. The results remained consistent after 1:1 propensity score matching with balancing of pre-treatment covariates and predominantly fell within international perioperative outcome benchmark values for patients having low-risk cohort characteristics.

Compared to previous studies using the SH in different patient cohorts, the current study observed similar morbidity rates, blood loss, and posthepatectomy outcomes. Accordingly, in the randomized controlled *Roc'n'Roll* trial, which compared robotic to laparoscopic hepatectomy for liver malignancies, predominantly the SH approach was used for robotic parenchymal transection with comparable findings [16]. In the present study, 43% of all resections were for benign tumors with most of these cases including alveolar echinococcosis posing a demanding resection due to the infiltrative growth pattern with proximity to vascular and biliary structures [35]. Nonetheless, a high rate of negative resection margins of 95% was recorded in our study. These findings indicate that the SH technique is safe and reproducible across different patient populations and in complex liver resections, with propensity score matching analysis not affecting these results. Furthermore, as opposed to existing concerns in literature, our data further indicates that sufficient hemostasis can be reached during surgery with basic robotic instruments even in complex liver resections [12]. A recent retrospective study compared two different robotic vessel sealing devices (SynchroSeal and VesselSealer) and reported no differences in severe morbidity, with rates ranging from 9 to 11% within 30 days after surgery. In this study, posthepatectomy outcomes, assessed according to ISGLS definitions, were also comparable. However, blood loss was slightly lower with the use of SynchroSeal compared to VesselSealer, with a difference of less than 50 mL [20]. In accordance, another retrospective study compared two parenchymal transection techniques using robotic devices versus a hybrid laparoscopic approach and detected no significant differences in perioperative outcomes [36]. In the study, the robotic parenchymal transection technique included a combination of bipolar forceps and vessel sealing device, whereas the laparoscopic method involved an ultrasonic dissection performed by a second bedside surgeon. Although posthepatectomy outcomes were not detailed according to the ISGLS definitions, the study showed less frequent severe complication rates (Clavien–Dindo grade III and higher), particularly < 4% within 30 days. Moreover, after 1:1 propensity score matching for 91 patients, the severe morbidity rate further dropped to 1%, however, with re-operation rates still at 3% which is obviously not in accordance with the definition of severe postoperative morbidity (including Clavien-Dindo grade III and higher). As opposed to these studies, however, we graded all complications that occurred within 90 days after surgery and further summarized outcomes with the comprehensive complication index. In addition, we included a substantially higher proportion of patients with ASA III of 65%, while rates for ASA III patients varied in both studies between 18 and 45%. Though in the highly selective subset of patients with low-risk demographic features (i.e., ASA $\le$ II, no major comorbidities or previous liver resections), the SH technique yielded excellent outcomes within the recently published benchmarks for robotic-assisted liver surgery [34]. Previous studies outlined ASA III as a significant risk factor of postoperative morbidity after liver surgery [37–41]. In our study, however, ASA III did not reach statistical significance with an OR 2.56 (95% CI 1.00–7.90; *p* = 0.068). Thus, substantial differences in patient cohorts, parenchymal transection techniques, perioperative standardizations, and definition of perioperative outcomes hamper side-by-side comparisons between studies in the absence of randomized trials.

This study has some limitations. First, this was a retrospective study conducted at a specialized high-volume center for minimally invasive liver surgery with experienced robotic liver surgeons [9, 16, 18, 35, 42], and the choice of transection technique was made intraoperatively at the surgeon’s discretion. Therefore, our results may not be comparable to others and prone to selection bias due to the retrospective study design and use of heterogeneous parenchymal transection techniques in the alternative hepatectomy group. To reduce bias, we used propensity score matching techniques in consecutive patients who had robotic hepatectomy. Secondly, there was a low proportion of major hepatectomies in the study, which is due to the institutional preference for parenchyma-sparing hepatectomy. Thirdly, long-term outcomes were not assessed in our study because the primary aim was to compare perioperative short-term results. In conclusion, the SH technique offers a safe, reproducible, and versatile option for parenchymal transection in robotic liver surgery. Future prospective randomized trials are required to confirm our findings and to enable a detailed comparison of the SH technique with other transection methods.

## Supplementary Information

Below is the link to the electronic supplementary material.

Supplementary file1 (DOCX 613 kb)

## Data Availability

The datasets generated and analyzed during the current study are available from the corresponding author on reasonable request.
