# Supplementary material for: Safety of robotic hepatic parenchymal transection using scissor hepatectomy and alternative techniques: a cohort study
Source: Surg Endosc. 2025 Nov 14;40(2):1235–43. doi: 10.1007/s00464-025-12382-0 (PMC12881050; doi:10.1007/s00464-025-12382-0)
Supplement: Supplementary file 1 — Supplementary file1 (DOCX 613 kb) [file 464_2025_12382_MOESM1_ESM.docx]

**SUPPLEMENTALS**

**Supplementary Figure 1:** The Comprehensive Complication Index scores of the study groups.

The Comprehensive Complication Index (CCI) stratified by the study groups is displayed. The mean scores (standard deviation) were 8.4 ± 18 versus 9.6 ± 21 in the SH and the AH group, respectively. There were no significant differences between the study groups (p = 0.64, Mann-Whitney-U Test). *Abbreviations: SH-group* = scissor hepatectomy group, *AH-group* = alternative techniques group.

**
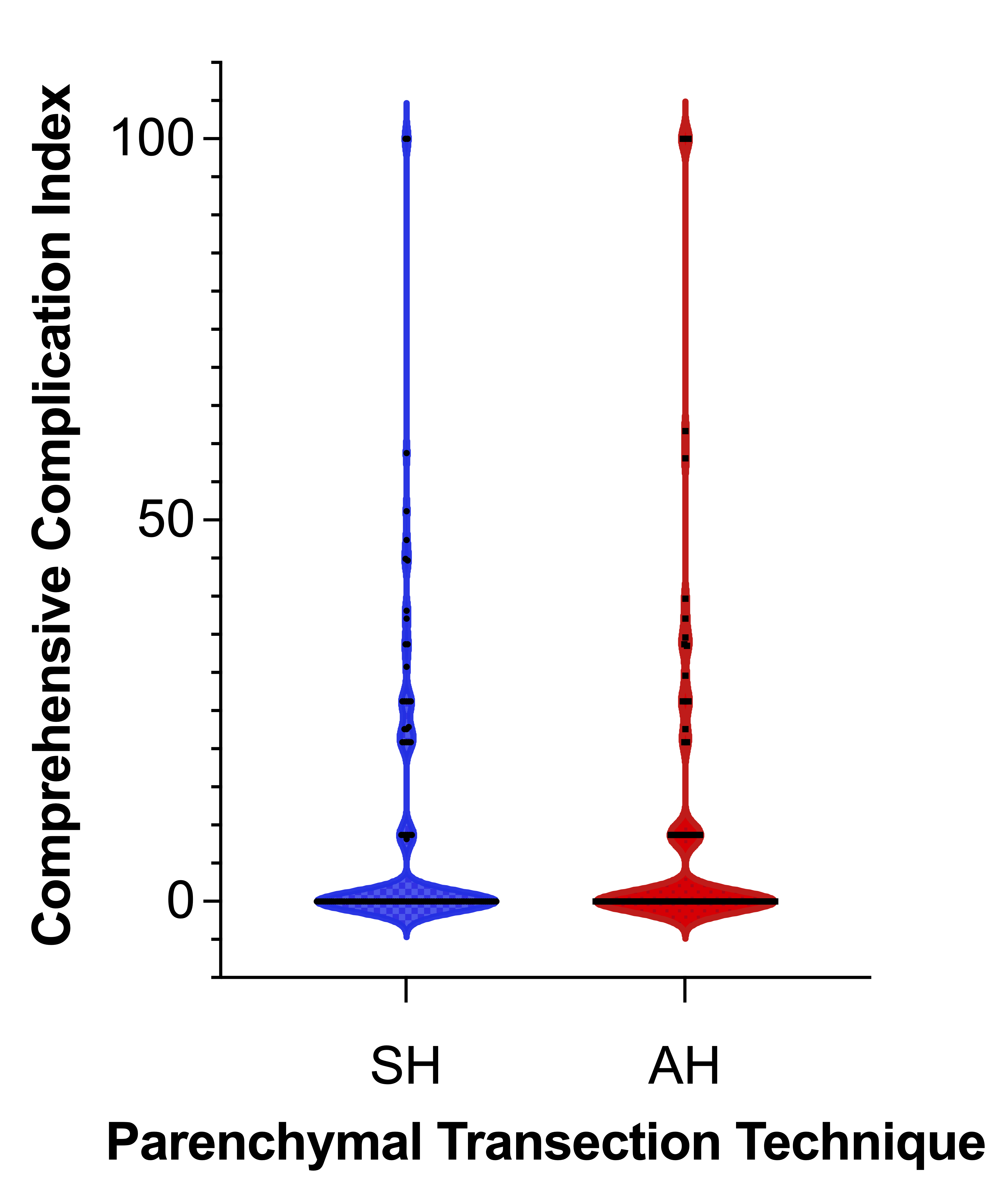
**

**Supplementary Table 1:** Baseline characteristics and perioperative outcomes after propensity-score matching

| **Characteristics** | **SH (n=71)** | **AH (n=71)** | **p value** | **SMD** |
| --- | --- | --- | --- | --- |
| Age, years | 64 (56-74) | 64 (53-69) | 0.218 | 0.202 |
| BMI, kg/m^2^ | 26 (23-28) | 26 (22-29) | 0.602 | 0.147 |
| Sex ratio, Male:Female | 36:35 | 37:34 | <0.99 | 0.028 |
| **ASA** |  |  | <0.99 | 0.079 |
| I | 2 (3) | 2 (3) |  |  |
| II | 22 (31) | 23 (32) |  |  |
| III | 44 (62) | 44 (62) |  |  |
| IV | 3 (4) | 2 (3) |  |  |
| Charlson comorbidity index | 4 (2-6) | 4 (2-6) | 0.884 | 0.040 |
| Cardiovascular comorbidities | 31 (44) | 30 (42) | <0.99 | 0.028 |
| Renal Insufficiency | 6 (9) | 2 (3) | 0.275 | 0.246 |
| Diabetes | 15 (21) | 11 (16) | 0.516 | 0.146 |
| Previous abdominal surgery | 38 (54) | 42 (59) | 0.612 | 0.114 |
| Liver steatosis | 24 (34) | 21 (30) | 0.719 | 0.091 |
| Liver cirrhosis | 12 (17) | 11 (16) | <0.99 | 0.038 |
| Child-Pugh class |  |  | 0.217 | 0.816 |
| Child A | 9 (13) | 11 (15) |  |  |
| Child B | 3 (4) | 0 |  |  |
| **Diagnosis** |  |  |  | 0.721 |
| HCC | 18 (25) | 13 (18) |  |  |
| CCC | 2 (3) | 8 (11) |  |  |
| Metastases  (CRLM and NCRLM) | 20 (28) | 22 (31) |  |  |
| Benign | 33 (46) | 28 (39) |  |  |
| Maximum lesion size diameter, mm | 43 (17-68) | 39 (25-57) | 0.969 | 0.143 |
| **Main hepatectomy procedure** |  |  | 0.794 | 0.275 |
| Non-anatomic resection | 33 (47) | 33 (47) |  |  |
| Segmentectomy (1 Segment) | 14 (20) | 16 (23) |  |  |
| Bisegmentectomy (2 Segments) | 11 (16) | 13 (18) |  |  |
| Multiple Segments (>2, e.g. Central hepatectomy) | 2 (3) | 3 (4) |  |  |
| (Extended) Right Hepatectomy | 7 (10) | 5 (7) |  |  |
| (Extended) Left Hepatectomy | 4 (6) | 1 (1) |  |  |
| **Extent of liver resection** |  |  | 0.470 | 0.162 |
| Major Hepatectomy (>3 Segments) | 12 (17) | 8 (11) |  |  |
| Minor Hepatectomy (1-3 Segments) | 59 (83) | 63 (89) |  |  |
| **Type of liver resection** |  |  | 0.376 | 0.241 |
| Anatomic | 27 (38) | 21 (30) |  |  |
| Non-Anatomic | 33 (47) | 33 (47) |  |  |
| Both | 11 (16) | 17 (24) |  |  |
| **Difficulty score (IWATE^a^)** | 6 (3-10) | 5 (4-7) | 0.900 | 0.082 |
| **Extrahepatic resection** | 12 (17) | 4 (6) | 0.060 | 0.362 |
| **Blood loss, mL** | 250  (125-500) | 400  (200-650) | **0.004** | 0.508 |
| **Operative time, min** | 205  (136-262) | 179  (149-268) | 0.933 | 0.008 |
| **Postoperative complications^b^** |  |  | 0.698 | 0.355 |
| Grade I | 4 (6) | 8 (11) |  |  |
| Grade II | 5 (7) | 4 (6) |  |  |
| Grade IIIa | 6 (9) | 4 (6) |  |  |
| Grade IIIb | 1 (1) | 2 (3) |  |  |
| Grade IVa | 0 | 2 (3) |  |  |
| Grade V | 2 (3) | 2 (3) |  |  |
| **Clinically relevant complications** |  |  |  |  |
| ≥ Grade IIIa | 9 (3) | 10 (15) | <0.99 | 0.041 |
| **Posthepatectomy bile leakage^c^** |  |  | <0.99 | 0.137 |
| Grade B | 2 (3) | 1 (1) |  |  |
| Grade C | 1 (1) | 2 (3) |  |  |
| **Posthepatectomy hemorrhage^c^** |  |  | 0.496 | 0.241 |
| Grade B | 0 | 1 (1) |  |  |
| Grade C | 0 | 1 (1) |  |  |
| **Posthepatectomy liver failure^c^** |  |  | <0.99 | 0.169 |
| Grade C | 1 (1) | 0 |  |  |
| **Length of stay, d** | 5 (4-7) | 5 (4-7) | 0.671 | 0.060 |
| **Readmission within 90 days after surgery** | 6 (9) | 5 (7) | <0.99 | 0.053 |
| **R Stage** |  |  | <0.99 | 0.038 |
| R0 | 68 (96) | 68 (96) |  |  |
| R1 | 3 (4) | 3 (4) |  |  |

Values are presented as median (interquartile range, IQR) or number (percentage), as appropriate. Values in bold, p<0.05.

^a^: Iwate difficulty scoring system is based on tumor location, tumor size, proximity to major vessels, liver function, and hand-assisted hybrid procedures.

^b^Postoperative complications are graded according to the Clavien–Dindo classification [30].

^c^Posthepatectomy complications as bile leakage, hemorrhage and liver failure are defined and graded according to the International Study Group of Liver Surgery (ISGLS) [32-34].

*Abbreviations:* *SH* = Scissor Hepatectomy; *AH* = alternative hepatectomy technique; *ASA* = American Society of Anesthesiologists; *BMI* = body mass index; *CCC* = cholangiocellular carcinoma; *CRLM* = colorectal liver metastases; *HCC* = hepatocellular carcinoma; *IQR* = interquartile range; *NCRLM* = non-colorectal liver metastases; *R stage* = resection margin status (R0: no residual tumor, R1: microscopic residual tumor); *SMD* = standardized mean difference.

**Supplementary Table 2:** Comparison of perioperative outcomes in low-risk patients with international benchmark outcomes stratified by extent of resection and IWATE difficulty

| **Benchmark characteristics** | **Minor**  **(n=22)** | **Major**  **(n=4)** | **IWATE^a^ 0-3**  **(n=8)** | **IWATE^a^ 4-6**  **(n=8)** | **IWATE^a^ 7-12**  **(n=10)** |
| --- | --- | --- | --- | --- | --- |
| Operative time, min | 140 (113 - 230) **BM:** $\boldsymbol{\leq}$ **294** | 345 (315 - 410)  **BM:** $\boldsymbol{\leq}$**437** | 123 (83 - 180) **BM:** $\boldsymbol{\leq}$ **234** | 130 (116 - 150) **BM:** $\boldsymbol{\leq}$ **354** | 281 (247 - 348) **BM:** $\boldsymbol{\leq}$ **357** |
| Blood loss, mL | 150 (100 - 300)  **BM:** $\boldsymbol{\leq}$ **100** | 500 (290 - 725)  **BM:** $\boldsymbol{\leq}$ **200** | 150 (88 - 225)  **BM:** $\boldsymbol{\leq}$ **83** | 100 (88 - 150)  **BM:** $\boldsymbol{\leq}$ **138** | 350 (260 - 650)  **BM:** $\boldsymbol{\leq}$ **200** |
| Intraoperative blood transfusion (u) | 0  **BM:** $\boldsymbol{\leq}$ **0** | 0  **BM:** $\boldsymbol{\leq}$ **0** | 0  **BM:** $\boldsymbol{\leq}$ **0** | 0  **BM:** $\boldsymbol{\leq}$ **0** | 0  **BM:** $\boldsymbol{\leq}$ **0** |
| Open conversion (%) | 0  **BM:** $\boldsymbol{\leq}$ **0** | 0  **BM:** $\boldsymbol{\leq}$ **0** | 0  **BM:** $\boldsymbol{\leq}$ **0** | 0  **BM:** $\boldsymbol{\leq}$ **3.5** | 0  **BM:** $\boldsymbol{\leq}$ **9.4** |
| Length of stay, d | 5 (3 - 7)  **BM:** $\boldsymbol{\leq}$ **5** | 5 (5 - 6)  **BM:** $\boldsymbol{\leq}$ **7** | 6 (3 - 9)  **BM:** $\boldsymbol{\leq}$ **5** | 3 (3 - 4)  **BM:** $\boldsymbol{\leq}$ **6** | 5 (5 - 6)  **BM:** $\boldsymbol{\leq}$ **7** |
| Any complication (%) | 9.1  **BM:** $\boldsymbol{\leq}$ **10.2** | 0  **BM:** $\boldsymbol{\leq}$ **31.2** | 12.5  **BM:** $\boldsymbol{\leq}$ **16.4** | 0  **BM:** $\boldsymbol{\leq}$ **16.5** | 10  **BM:** $\boldsymbol{\leq}$ **21.5** |
| Clavien-Dindo ≥ Grade IIIa^b^ (%) | 0  **BM:** $\boldsymbol{\leq}$ **5.3** | 0  **BM:** $\boldsymbol{\leq}$ **17.4** | 0  **BM:** $\boldsymbol{\leq}$ **2.2** | 0  **BM:** $\boldsymbol{\leq}$ **5.9** | 0  **BM:** $\boldsymbol{\leq}$ **9.5** |
| Median CCI | 0  **BM:** $\boldsymbol{\leq}$ **0** | 0  **BM:** $\boldsymbol{\leq}$ **0** | 0  **BM:** $\boldsymbol{\leq}$ **0** | 0  **BM:** $\boldsymbol{\leq}$ **0** | 0  **BM:** $\boldsymbol{\leq}$ **0** |
| Relaparotomy rate (%) | 0  **BM:** $\boldsymbol{\leq}$ **0** | 0  **BM:** $\boldsymbol{\leq}$ **0** | 0  **BM:** $\boldsymbol{\leq}$ **0** | 0  **BM:** $\boldsymbol{\leq}$ **0.2** | 0  **BM:** $\boldsymbol{\leq}$ **0** |
| Readmission rate (%) | 0  **BM:** $\boldsymbol{\leq}$ **0** | 0  **BM:** $\boldsymbol{\leq}$ **0** | 0  **BM:** $\boldsymbol{\leq}$ **0** | 0  **BM:** $\boldsymbol{\leq}$ **4.2** | 0  **BM:** $\boldsymbol{\leq}$ **5.0** |
| Mortality (%) | 0  **BM:** $\boldsymbol{\leq}$ **0** | 0  **BM:** $\boldsymbol{\leq}$ **0** | 0  **BM:** $\boldsymbol{\leq}$ **0** | 0  **BM:** $\boldsymbol{\leq}$ **0** | 0  **BM:** $\boldsymbol{\leq}$ **0** |
| Posthepatectomy liver failure B/C^c^ (%) | 0  **BM:** $\boldsymbol{\leq}$ **0** | 0  **BM:** $\boldsymbol{\leq}$ **0** | 0  **BM:** $\boldsymbol{\leq}$ **0** | 0  **BM:** $\boldsymbol{\leq}$ **0** | 0  **BM:** $\boldsymbol{\leq}$ **0.3** |
| Posthepatectomy bile leakage B/C^c^ (%) | 0  **BM:** $\boldsymbol{\leq}$ **1.1** | 0  **BM:** $\boldsymbol{\leq}$ **0** | 0  **BM:** $\boldsymbol{\leq}$ **0** | 0  **BM:** $\boldsymbol{\leq}$ **1.8** | 0  **BM:** $\boldsymbol{\leq}$ **7.1** |
| Textbook Outcome (%) | 100  **BM:** $\boldsymbol{\geq}$ **93** | 100  **BM:** $\boldsymbol{\geq}$ **82** | 100  **BM:** $\boldsymbol{\geq}$ **84.2** | 100  **BM:** $\boldsymbol{\geq}$ **76.2** | 100  **BM:** $\boldsymbol{\geq}$ **67.9** |
| R1 resection (%) | 0  **BM:** $\boldsymbol{\leq}$ **2.2** | 0  **BM:** $\boldsymbol{\leq}$ **9.2** | 0  **BM:** $\boldsymbol{\leq}$ **5.7** | 0  **BM:** $\boldsymbol{\leq}$ **5.7** | 0  **BM:** $\boldsymbol{\leq}$ **14.1** |

Values are presented as median (interquartile range, IQR) or number (percentage), as appropriate. Benchmark outcomes and values were previously published in a low-risk cohort by Li et al. 2025. Minor hepatectomy was defined as resection of ≤3 anatomical liver segments, whereas major hepatectomy was defined as resection of >3 segments.

^a^: Iwate difficulty scoring system is based on tumor location, tumor size, proximity to major vessels, liver function, and hand-assisted hybrid procedures. ^b^Postoperative complications are graded according to the Clavien–Dindo classification^2^. ^c^Posthepatectomy complications as bile leakage, hemorrhage and liver failure are defined and graded according to the International Study Group of Liver Surgery (ISGLS) *Abbreviations:* *BM* = Benchmark; *CCI =* Comprehensive Complication Index*; R stage* = resection margin status (R1: microscopic residual tumor).

**Supplementary Table 3:** Multivariable logistic regression analysis of clinically relevant postoperative complications.

|  | **OR** | **95% - CI** | **p value** |
| --- | --- | --- | --- |
| **Age ≥ 64 years** | 1.23 | 0.49 – 3.06 | 0.663 |
| **ASA ≥ III** | 2.22 | 0.72 – 6.82 | 0.163 |
| **Blood loss ≥ 500 mL** | 1.64 | 0.71 – 3.78 | 0.247 |
| **IWATE^a^ ≥ 6** | 1.25 | 0.59 – 3.12 | 0.638 |
| **Major Hepatectomy^b^** | 0.52 | 0.18 – 1.51 | 0.231 |

All variables with a univariable p-value <0.2 were included in the multivariable logistic regression model.

^a^: Iwate difficulty scoring system is based on tumor location, tumor size, proximity to major vessels, liver function, and hand-assisted hybrid procedures.

^b^: Defined as resection of more than three anatomical liver segments.

Abbreviations: *OR* Odds ratio, *CI* Confidence interval, *ASA* American Society of Anesthesiologists

**Supplementary File: STROBE Checklist**

STROBE Statement—checklist of items that should be included in reports of observational studies

|  | Item No. | Recommendation | Page  No. | Relevant text from manuscript |
| --- | --- | --- | --- | --- |
| **Title and abstract** | 1 | (*a*) Indicate the study’s design with a commonly used term in the title or the abstract | 1 | “A Cohort Study” |
|  |  | (*b*) Provide in the abstract an informative and balanced summary of what was done and what was found | 2 | See “Abstract” |
| Introduction | | | |  |
| Background/rationale | 2 | Explain the scientific background and rationale for the investigation being reported | 3 | “Introduction” |
| Objectives | 3 | State specific objectives, including any prespecified hypotheses | 3 | “… was to analyze the postoperative outcomes comparing SH with …” |
| Methods | | | |  |
| Study design | 4 | Present key elements of study design early in the paper | 4 | “This cohort study…” |
| Setting | 5 | Describe the setting, locations, and relevant dates, including periods of recruitment, exposure, follow-up, and data collection | 4 | “All patients undergoing robotic hepatectomy for benign or malignant liver lesions from November 2020 through December 2024 at the University Hospital Ulm…” |
| Participants | 6 | (*a*) *Cohort study*—Give the eligibility criteria, and the sources and methods of selection of participants. Describe methods of follow-up  *Case-control study*—Give the eligibility criteria, and the sources and methods of case ascertainment and control selection. Give the rationale for the choice of cases and controls  *Cross-sectional study*—Give the eligibility criteria, and the sources and methods of selection of participants | 4,5 | “…from a prospectively maintained database.” “Cases were excluded if…” “…the 90-day morbidity rate..” |
|  |  | (*b*) *Cohort study*—For matched studies, give matching criteria and number of exposed and unexposed  *Case-control study*—For matched studies, give matching criteria and the number of controls per case | 6 | "Patients who underwent SH were matched 1:1 with AH patients using nearest neighbor matching algorithm" |
| Variables | 7 | Clearly define all outcomes, exposures, predictors, potential confounders, and effect modifiers. Give diagnostic criteria, if applicable | 5 | see Methods - Outcomes |
| Data sources/ measurement | 8* | For each variable of interest, give sources of data and details of methods of assessment (measurement). Describe comparability of assessment methods if there is more than one group | *5* |  |
| Bias | 9 | Describe any efforts to address potential sources of bias | 6 | Propensity scores for receiving SH were calculated via multivariate logistic regression using pre-treatment variables |
| Study size | 10 | Explain how the study size was arrived at | 4 | “All patients undergoing… from November 2020 through December 2024…” |

| Quantitative variables | 11 | Explain how quantitative variables were handled in the analyses. If applicable, describe which groupings were chosen and why | 5,7 | “…117 in the SH and 90 patients in the AH group.” |
| --- | --- | --- | --- | --- |
| Statistical methods | 12 | (*a*) Describe all statistical methods, including those used to control for confounding | 5,6 | See Methods - Statistics |
|  |  | (*b*) Describe any methods used to examine subgroups and interactions | 5,6 | See Methods - Statistics |
|  |  | (*c*) Explain how missing data were addressed | - | See Methods - Statistics |
|  |  | (*d*) *Cohort study*—If applicable, explain how loss to follow-up was addressed  *Case-control study*—If applicable, explain how matching of cases and controls was addressed  *Cross-sectional study*—If applicable, describe analytical methods taking account of sampling strategy |  |  |
|  |  | (*e*) Describe any sensitivity analyses | 5,6 | See Methods - Statistics |
| Results | | | | |
| Participants | 13* | (a) Report numbers of individuals at each stage of study—eg numbers potentially eligible, examined for eligibility, confirmed eligible, included in the study, completing follow-up, and analysed | 7 | “Of 243 robotic …, a total of 207…” |
|  |  | (b) Give reasons for non-participation at each stage | 4,7,  Flow Chart | “Cases were excluded if…” |
|  |  | (c) Consider use of a flow diagram | Flow Chart | See Flow Chart |
| Descriptive data | 14* | (a) Give characteristics of study participants (eg demographic, clinical, social) and information on exposures and potential confounders | 7 | “Most patients were male …”, Table 1 |
|  |  | (b) Indicate number of participants with missing data for each variable of interest | - |  |
|  |  | (c) *Cohort study*—Summarise follow-up time (eg, average and total amount) | 8 | “within 90 days after surgery” |
| Outcome data | 15* | *Cohort study*—Report numbers of outcome events or summary measures over time | *8* | “Overall, a total of 61 patients experienced postoperative complications…”, Table 3 |
|  |  | *Case-control study—*Report numbers in each exposure category, or summary measures of exposure | *-* |  |
|  |  | *Cross-sectional study—*Report numbers of outcome events or summary measures | *-* |  |
| Main results | 16 | (*a*) Give unadjusted estimates and, if applicable, confounder-adjusted estimates and their precision (eg, 95% confidence interval). Make clear which confounders were adjusted for and why they were included | 8 | Figure 2: Forest plots |
|  |  | (*b*) Report category boundaries when continuous variables were categorized | 5,7,8 | Methods - Outcomes |
|  |  | (*c*) If relevant, consider translating estimates of relative risk into absolute risk for a meaningful time period |  |  |

| Other analyses | 17 | Report other analyses done—eg analyses of subgroups and interactions, and sensitivity analyses | 8 | Forest plots |
| --- | --- | --- | --- | --- |
| Discussion | | | | |
| Key results | 18 | Summarise key results with reference to study objectives | 9,12 | “Our findings revealed no differences in perioperative morbidity…”, “In conclusion, SH…” |
| Limitations | 19 | Discuss limitations of the study, taking into account sources of potential bias or imprecision. Discuss both direction and magnitude of any potential bias | 12 | “This study has somoe limitations…” |
| Interpretation | 20 | Give a cautious overall interpretation of results considering objectives, limitations, multiplicity of analyses, results from similar studies, and other relevant evidence | 12 | “Future prospective randomized trials are required to confirm our findings…” |
| Generalisability | 21 | Discuss the generalisability (external validity) of the study results | 12 | “…conducted at a specialized high-volume center…” “Therefore, our results may not be comparable..” |
| Other information | |  | | |
| Funding | 22 | Give the source of funding and the role of the funders for the present study and, if applicable, for the original study on which the present article is based | 1 | “This research did not receive any specific grants…” |

*Give information separately for cases and controls in case-control studies and, if applicable, for exposed and unexposed groups in cohort and cross-sectional studies.

**Note:** An Explanation and Elaboration article discusses each checklist item and gives methodological background and published examples of transparent reporting. The STROBE checklist is best used in conjunction with this article (freely available on the Web sites of PLoS Medicine at http://www.plosmedicine.org/, Annals of Internal Medicine at http://www.annals.org/, and Epidemiology at http://www.epidem.com/). Information on the STROBE Initiative is available at www.strobe-statement.org.
